# Supplementary material for: Uprooting defects to enable high-performance III–V optoelectronic devices on silicon
Source: Nat Commun. 2019 Sep 20;10:4322. doi: 10.1038/s41467-019-12353-9 (PMC6754402; doi:10.1038/s41467-019-12353-9)
Supplement: Supplementary file 1 — Supplementary Information [file 41467_2019_12353_MOESM1_ESM.pdf]

SUPPLEMENTARY INFORMATION

Uprooting defects to enable high-performance III–V optoelectronic devices on Si

Bioud et al.

Supplementary Note 1: Nanovoids formation induced by Ostwald ripening

Supplementary Figure 1 shows different void of different shapes stabilized following heat treatment of the porous Ge layer. The atoms diffuse from the larger curvature surface toward the lower curvature surface down a gradient potential following to Rayleigh instabilities [1]. Depending on annealing temperature) random pores obtained after electrochemical etching become spherical voids beginning from 350°C (Supplementary Figure 1a). The neighboring voids are then coalesced to form larger ones at 450°C, as shown in Figure 1b. Keeping activation energy, surface atoms of spherical voids form faced voids that are minimizing the surface energy by favoring the growth of low-energy facets (see Supplementary Figure 1c,d). Faced voids start to dominate, from 600°C. The measured angle between the two prominent orientations is in perfect agreement with the angle value of ~54° between the lowest surface energy (100) and (111) oriented facets in crystalline Ge. High angle annular dark field scanning transmission electron microscopy (HAADF-STEM) micrograph from the facet void is presented in Figure 1e and shows a monocrystalline quality of the Ge after annealing at 600°C. Structural analysis by µ-Raman of as-prepared porous- and annealed Ge/Si is presented in Supplementary Figure 1f. The spectral position, the width and the shape of the Raman peak of as-prepared PGe/Si are quite different from the well-known Ge bulk position at 300 cm^{-1}. These changes are attributed to the quantum confinement of phonons at low crystal size confirming the nanostructuration of the Ge layer [2-4]. However, the peak from the annealed PGe/Si shows the disappearance of the asymmetric form, a decrease of the width and a coincidence with the spectrum of bulk Ge, indicting the restoring of the bulk order in the voided Ge/Si substrate.

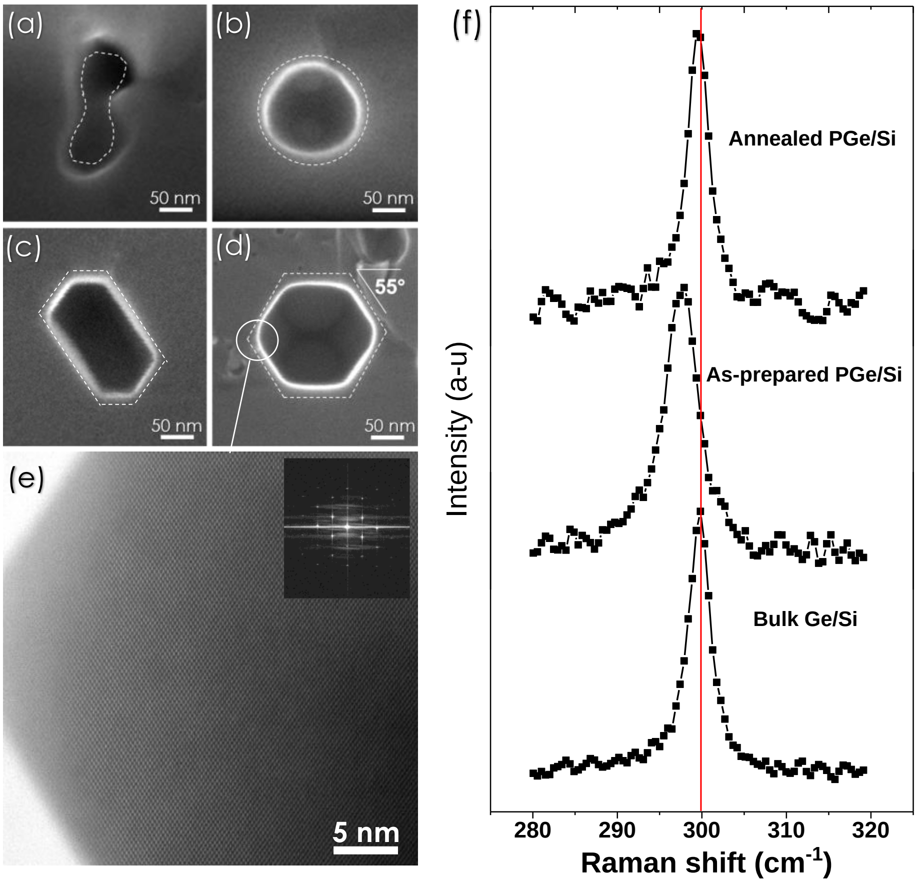

Supplementary Figure 1. Voids of different shapes observed during the annealing of Ge/Si layer formed following to the mass transport mechanism: (a) at 350°C, the beginning of the coalescence of two nearest pores given elongated voids (b) at 450°C, spherical voids start to dominate and (c,d) from 600°C, faced voids can be observed. The heat treatment was performed in hydrogen ambient during 10 min. The measured angle is in perfect agreement with the angle value of ~54° between the (100) and (111) oriented facets. (e) High angle annular dark field scanning transmission electron microscopy HAADF-STEM micrograph from (d) showing a monocrystalline quality of the Ge after annealing at 600°C. (f) Evolution of Raman spectra at the porosification and annealing stpes. The Raman spectra of bulk Ge/Si is also presented for comparison.

Supplementary Note 2: FFT analyses of the NVS interface

Supplementary Figure 2a-c shows EDX mapping of the NVS revealing an unclear, unsharp, Ge/Si interface, probably due to a mixed reorganization of the porous Si and Ge atoms at the interface during thermal annealing. Supplementary Figure 2d shows an enlarged image of the region near the Ge/Si interface by HRTEM fabricated following 110 zone axis. A fast Fourier transform (FFT) image of Supplementary Figure 2d is given from different zones, which reveals clearly the monocrystalline quality of the Ge layer after undergone a strong perturbation during the electrochemical etching and annealing. The amorphous zone appear in the void zone,

probably due to the amorphisation of the void wall during the TEM sample milling or due the superposition of diffraction from the different crystalline material separated by voids. The diffraction spots from the interface are divided into paired separate spots that are indicated by two white lines, which correspond with those of Si and Ge crystals, confirming the same crystallographic orientation as the parent Si wafer. Supplementary Figure 2e shows an inverse FFT image (IFFT) obtained through the (11-1) spot in Supplementary Figure 2d. Lines in the numerical Moiré images correspond with lattice planes. From the interface, the edge component *b* of the 60° dislocation in the Si is inclined at ~54° to the interface, and only one extra half-plane exists, marked by an arrow. The period lines in Supplementary Figure 2f and 2g are 0.33 nm and 0.32 nm, correspond to the Ge and Si {111} interplanar spacing, respectively.

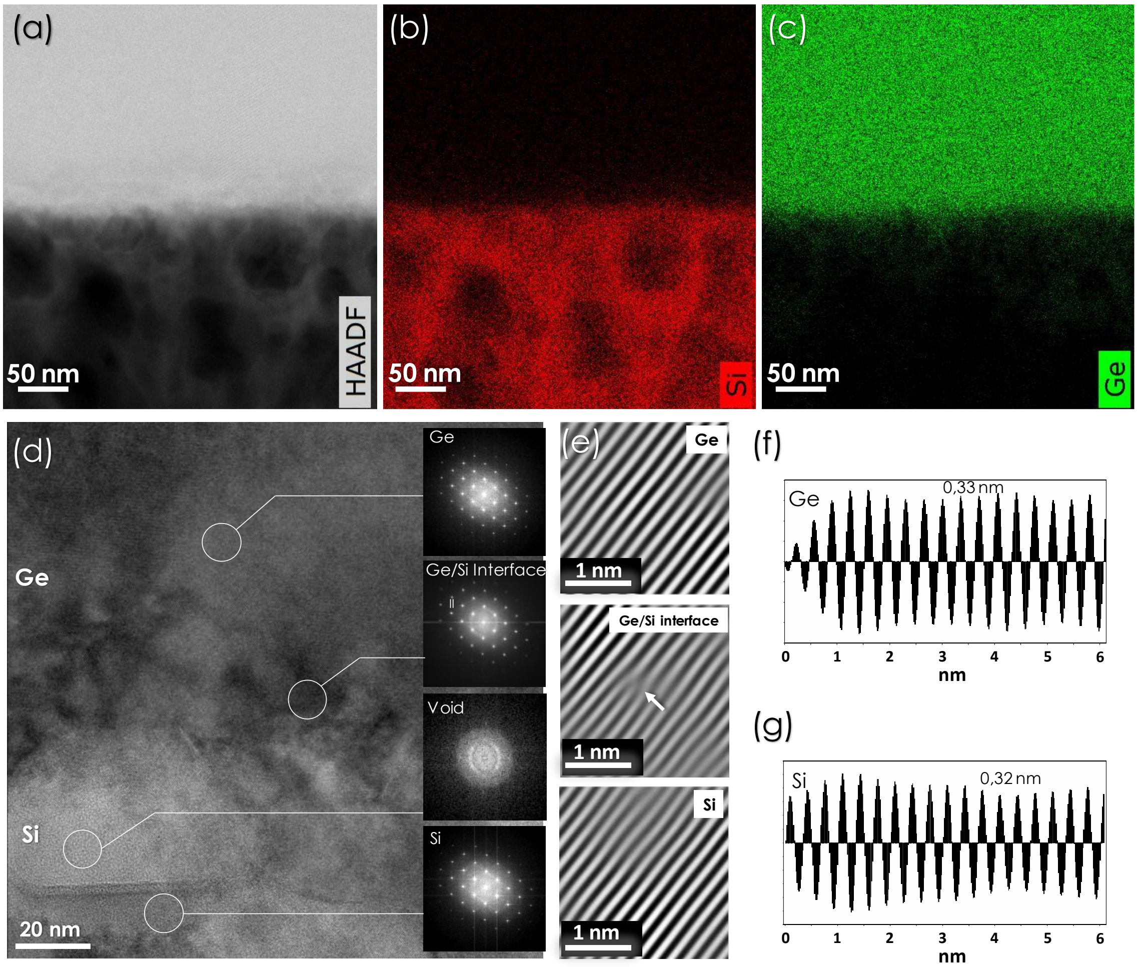

Supplementary Figure 2. Energy-dispersive X-ray spectroscopy (EDX) mapping of the nanovoid based Si virtual substrate. (a) Cross section scanning transmission electron microscopy (STEM) image and the EDX element maps for (b) Si and (c) Ge elements. (d) High-resolution transmission electron microscopy (TEM) image from the interface of the Ge/Si layer and Fast Fourier transform (FFT) patterns (inset) of the marked Ge/Si heterostructure. (e-g) Numerical moiré of (11-1) plane from the Ge layer, and the Si substrate and their intensity profiles illustrating the Ge {111} and the Si {111} inter-planar spacing of ~3.3 Å and ~3.2 Å, respectively.

Supplementary Note 3: Determination of TDD by etch-pit-density (EPD)

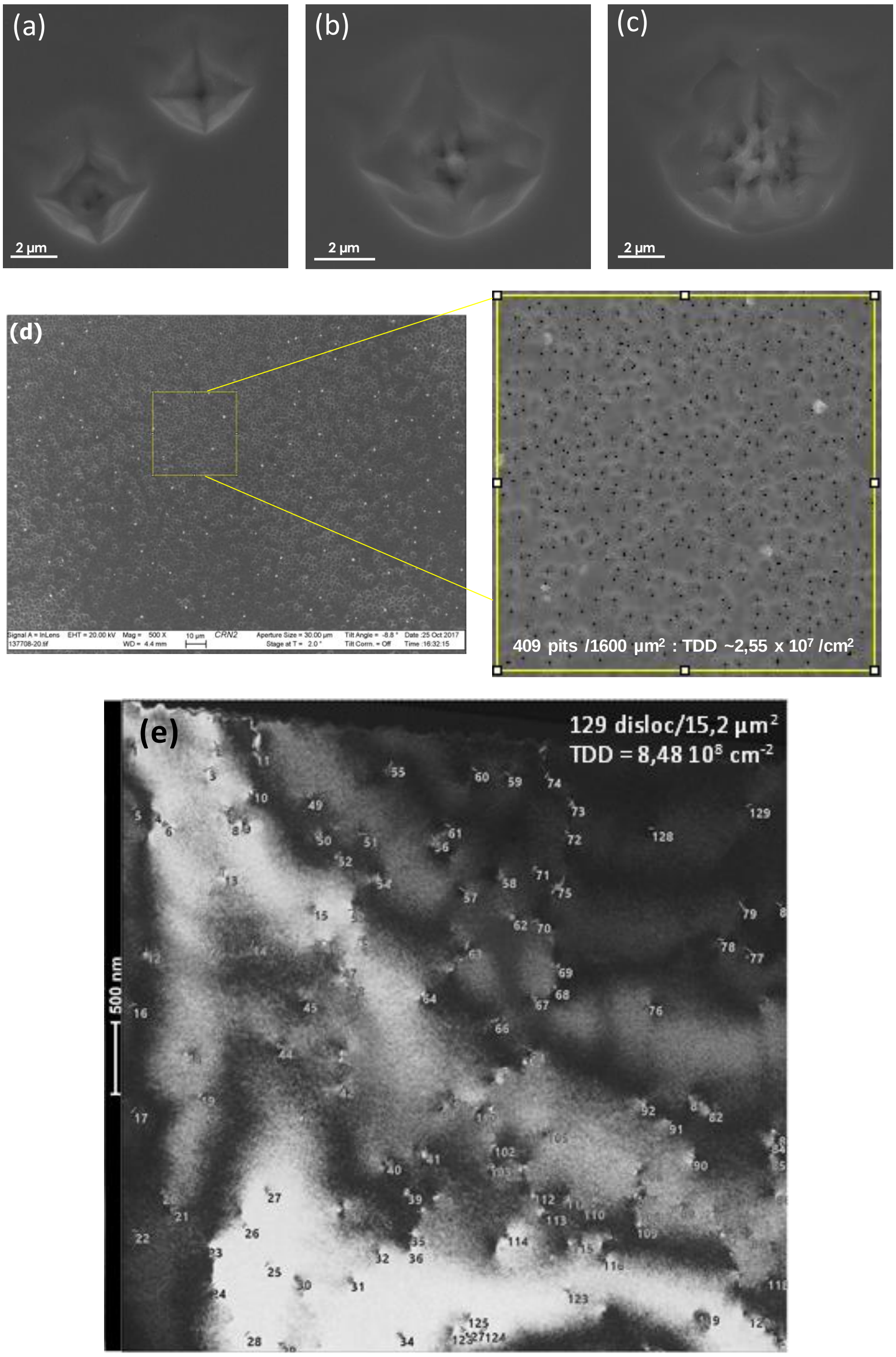

Supplementary Figure 3. Scanning electron microscopy (SEM) images of different shapes of etch pits (e,b,c) and the computation of etch pit density using ImageJ software (d) showing that etch-pits may only be revealing a fraction of the threading dislocations (TDs) contrary to plan-view transmission electron microscopy (TEM) (e).

Supplementary Note 4: Optimization of the chemical mechanical polishing (CMP) process

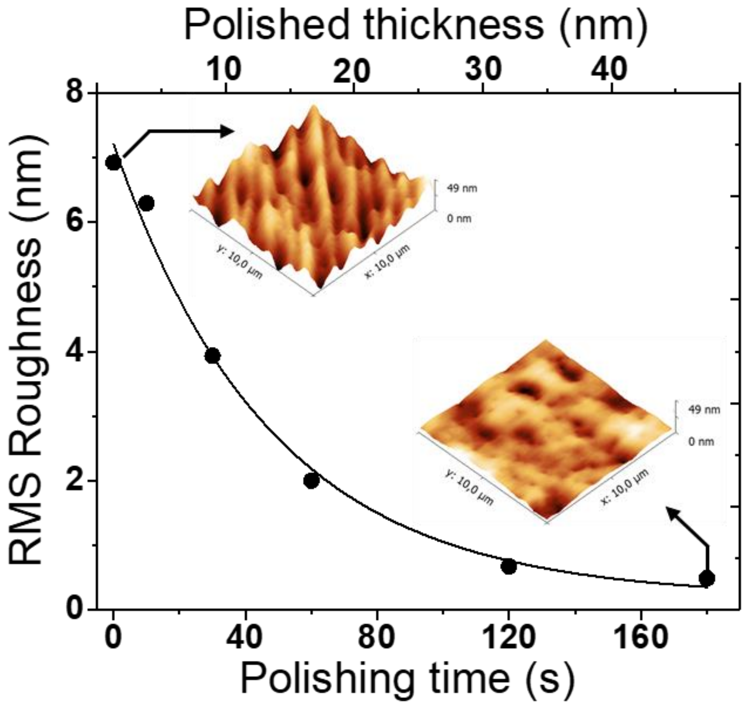

Supplementary Figure 4. Root Mean Square (RMS) surface roughness and polished thickness of Ge/Si dependence of Chemical-mechanical polishing (CMP) polishing time, using a mixture of commercial CMP slurries, Deionized (DI) water dilution, and 1 wt% H_2O_2.

Supplementary Note 5: Monte Carlo simulation of cathodoluminescence (CL) generated from GaAs/Ge

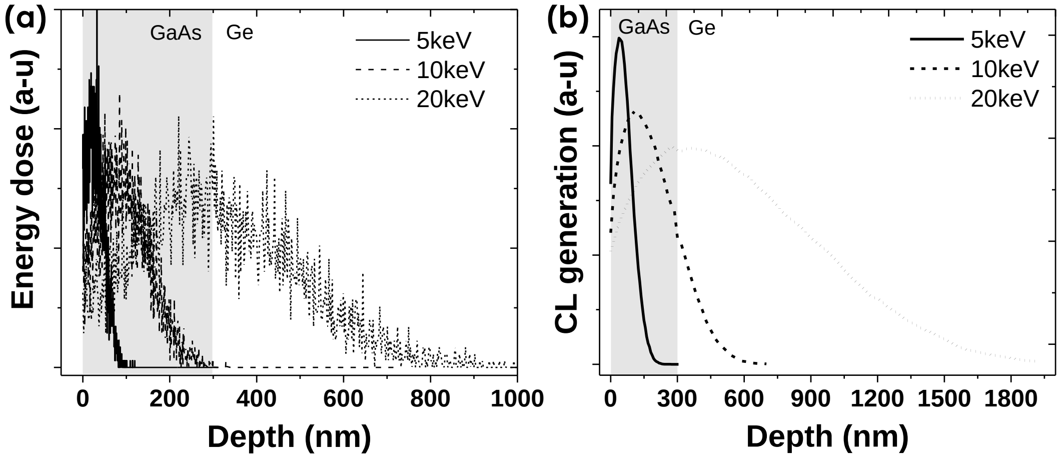

Supplementary Figure 5. (a) Simulated total energy-dose profiles for a 300 nm GaAs epilayer grown on a Ge substrate calculated using the Monte Carlo simulation CASINO and (b) Simulated generation profiles for the measured near-edge cathodoluminescence (CL) signal for 5 keV, 10 keV and 20 keV beam energies.

Supplementary References:

[1] L. Rayleigh, "On the Instability of Jets," *Proc. London Math. Soc.*, (1878).

[2] A. Boucherif, G. Beaudin, V. Aimez, and R. Arès, "Mesoporous germanium morphology transformation for lift-off process and substrate re-use," *Appl. Phys. Lett.*, 102, 011915, (2013).

[3] M. Isaiev *et al.*, "Thermal conductivity of meso-porous germanium," *Appl. Phys. Lett.*, 102, 011915, (2014).

[4] S. Tutashkonko *et al.*, "Mesoporous Germanium formed by bipolar electrochemical etching," *Electrochim. Acta*, 88, 256–262, (2013).
